# Application of bacteria and bacteriophage cocktails for biological control of houseflies

**DOI:** 10.1186/s13071-023-06082-8

**Published:** 2024-01-17

**Authors:** Kexin Zhang, Shumin Wang, Ying Li, Yansong Yin, Xinyu Zhang, Qian Zhang, Xinxin Kong, Wenjuan Liu, Dawei Yao, Ruiling Zhang, Zhong Zhang

**Affiliations:** 1 Hospital for Skin Diseases, Shandong First Medical University, Jinan, China; 2 Shandong Provincial Institute of Dermatology and Venereology, Shandong Academy of Medical Sciences, Jinan, China; 3 Collaborative Innovation Center for the Origin and Control of Emerging Infectious Diseases, Shandong First Medical University and Shandong Academy of Medical Sciences, Taian, China; 4 School of Clinical and Basic Medical Science, Shandong First Medical University and Shandong Academy of Medical Sciences, Jinan, China; 5 School of Life Science, Shandong First Medical University and Shandong Academy of Medical Sciences, Taian, China; 6 Shandong Institute of Endocrine and Metabolic Diseases, Shandong First Medical University, Jinan, Shandong China; 7 Department of Endocrinology, Shandong Provincial Hospital Affiliated to Shandong First Medical University, Jinan, China; 8 Department of Laboratory Medicine, Sun Yat-Sen Memorial Hospital, Sun Yat-Sen University, Shanwei, China; 9 School of Life Science, Weifang Medical University, Weifang, China; 10 Medical Science and Technology Innovation Center, The First Affiliated Hospital of Shandong First Medical University, Jinan, China

**Keywords:** Intestinal flora, Housefly, Bacteria/phage cocktails, Vector control

## Abstract

**Background:**

Houseflies, *Musca domestica* L., are an ubiquitous pest that can transmit numerous diseases and threaten human health. Increasing insecticide resistance shown by houseflies necessitates the develop new control alternatives. The housefly gut is densely colonized with microorganisms that interact with each other dynamically and benefit the host’s health. However, the impact of multiple symbiotic bacteria on the composition of housefly gut microbiota and the host’s activities remains unclear.

**Methods:**

We isolated and cultured 12 bacterial species from the intestines of housefly larvae. We also isolated seven bacteriophages to precisely target the regulation of certain bacterial species. Using 16S rRNA high-throughput gene sequencing, we analyzed the bacterial diversity after orally administering bacteria/phage cocktails to houseflies.

**Results:**

Our results showed that larval growth was promoted, the abundance of beneficial bacteria, such as *Klebsiella* and *Enterobacter*, was increased and the abundance of harmful bacteria, such as *Providencia*, *Morganella* and *Pseudomonas,* was decreased in housefly larvae fed with the beneficial bacteria cocktail. However, oral administration of both beneficial and harmful bacterial phage cocktails inhibited larval growth, probably due to the drastic alteration of gut flora. Untargeted metabolomics using liquid chromatography–mass spectrometry showed that disturbances in gut microbiota changed the larval metabolite profiles. Feeding experiments revealed that disrupting the intestinal flora suppressed the beneficial bacteria and increased the harmful bacteria, causing changes in the metabolites and inhibiting larval growth.

**Conclusions:**

Based on our results, bacteria/phage cocktails are effective tools for regulating the intestinal flora of insects and have a high potential as a biological control agent for incorporation into an integrated pest management program.

**Graphical abstract:**

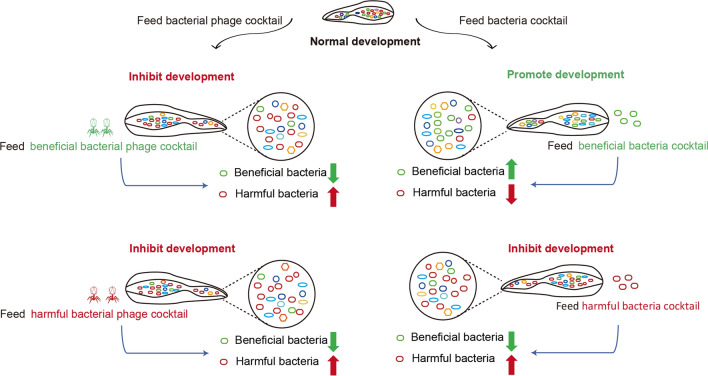

**Supplementary Information:**

The online version contains supplementary material available at 10.1186/s13071-023-06082-8.

## Background

The insect gut contains complex bacterial communities that are crucial for their development and influence the host’s physiology [1]. Some beneficial bacteria provide nutrients that enhance the host’s development, reproduction [2], immunity [3] and longevity [4]. Symbiotic bacteria improve the host’s health by providing resistance against pathogenic fungi [5]. However, certain bacterial pathogens can infect insects [6] via transmission or interaction with pathogenic fungi [7]. Studies have revealed that inoculating housefly larvae with a mixture of *Escherichia coli* and *Staphylococcus aureus* induced several of the genes regulating the insect’s innate immunity. Dynamic gene expression changes observed in bacteria-infected houseflies enabled an in-depth analysis of *Musca domestica’s* immune system [8, 9]. *Serratia marcescens* strain Sm_YN3, found in the intestines of the *Anopheles* mosquito, was found to attenuate *Plasmodium* parasites by activating the Toll-like receptors [10]. The intestinal microbes in insects can affect infection by pathogens. The intestinal microbiome of *Anopheles gambiae* has been shown to produce antiparasitic effectors that suppress *Plasmodium* [11]. The symbiotic bacteria in mosquito intestines can limit viral infection by initiating host immune surveillance and secreting microbial metabolites [12, 13]. However, a recent study showed that *Talaromyces* (Tsp_PR) fungus in the *Aedes aegypti* gut increased the host’s tolerance to dengue virus infection by downregulating genes for digestive enzymes and trypsin activity in the mosquito’s intestines [14]. These findings indicate the complex roles played by the gut commensal microbiome in arboviral infection and transmission.

The housefly *M. domestica* L. (Diptera: Muscidae) is an ubiquitous cosmopolitan pest. Houseflies which serve as mechanical vectors can threat human and animal health by transmitting a number of human and animal disease-causing pathogens [15–17]. Chemical insecticides have been widely used to control *M. domestica* [18], but excessive use of such synthetic chemical insecticides may create potential risks for both the environment and human health and contribute to the emergence of insecticide resistance [19–21]. To minimize negative health and environmental effects, there is a need for new alternative and selective strategies and resources for pest and vector insect control. Biological control agents (BCAs) are generally seen as a safer alternative of pest management. Entomopathogenic fungi are promising BCAs for housefly management. Some known species of entomopathogenic fungi, such as *Beauveria bassiana * and* Metarhizium anisopliae* sensu lato have been investigated for use in the control of houseflies and have shown promise for housefly management [22–24]. Most microbial control research to date has focused on entomopathogenic fungi, with limited consideration given to bacterial pathogens [25]. Several genera of bacteria have been used as bio-pesticides in pest control. Bacteria of the genus *Bacillus* are promising pathogens for biological control of targeted insects [26]. *Lysinibacillus sphaericus* has been used effectively for the biological control of various mosquito species by producing a toxin [27]. In our previous research, *S. marcescens* and *Pseudomonas aeruginosa* were found to affect larval development in houseflies by inhibiting the growth of beneficial bacteria and reducing larval humoral immunity [28, 29]. Given that various mixed bacterial biological agents have received little attention for housefly control, the goal of this study was to examine the effects of bacteria and bacteriophage cocktails on the housefly as alternative biological control agents.

In the host insects, changes in gut microbiota affect multiple signaling pathways and metabolic processes, such as bile acid biosynthesis, the urea cycle, choline metabolism [30], as well as certain metabolites. It has been reported that the gut microbiota can regulate bile acid metabolism through bacterial deamidation, dehydroxylation, oxidation and epimerization reactions [31]. Similarly, metabolites influence insect development by providing important nutrients for extending the lifespan or enhancing the host’s resistance to pathogenic bacteria [32–34]. However, as most studies on the intestinal bacteria of insects have focused on a single bacterium, the effect of multiple bacterial species is unclear. Moreover, most such studies mainly performed “total removal and replenishment,” which greatly altered the nontargeted gut bacteria. Therefore, novel strategies need to be developed to modulate the targeted bacteria more precisely and rationally in the gut microbiota. Previously, we established a phage-targeted method to reduce *Enterobacter hormaechei* in the housefly larval gut. We also preliminarily explored the effects of *E. hormaechei* on larval growth and disturbance of the intestinal flora [35]. Our previous findings showed that phages can be used to precisely knock down the susceptible bacteria in the housefly gut microbiota.

 In the present study, we used bacteriophages to specifically knock down susceptible species to regulate gut microbiota. We orally administered a bacterial/phage cocktail to evaluate its effects on the housefly larval gut microbiota and larval growth. Using 16S ribosomal RNA (rRNA) high-throughput gene sequencing and untargeted metabolomics, we analyzed the bacterial diversity and metabolites. We used five beneficial and two harmful gut bacterial strains, and their corresponding phages to investigate the effects of adding and removing bacterial mixtures on larval growth. We also analyzed the changes in gut microbiota, metabonomics and phenoloxidase activity. Our results showed that harmful bacterial and phage cocktails can be used as a biological control for housefly larvae by regulating intestinal microecology. We speculate that the effect of oral administration of a single bacteriophage on the larval gut microbiota is weaker than that of bacteriophage cocktails, thereby increasing harmful bacteria and inhibiting larval growth. Our work provides insight into the biological control of pests.

## Methods

### Animal and microbial strains

A housefly (*M. domestica*) colony from the Laboratory of Vector and Insect Diseases of Shandong First Medical University, reared since 2005 in the Laboratory [36], was used in this study.

Beneficial bacteria, including *E. hormaechei* EhX,* Klebsiella pneumoniae* KX,* Acinetobacter bereziniae* Ab,* Enterobacter cloacae* Ec,* Lysinibacillus fusiformis* Lf and* Bacillus safensis* Bs, harmful bacteria, including *P. aeruginosa* Y12,* Providencia stuartii* Ps and *Providencia vermicola* Pv, and neutral bacteria, including *Lactococcus lactis* Ll, were isolated as described previously [28]. *S. marcescens* Sm and *Enterococcus faecalis* Ef were isolated under aerobic and facultative anaerobic conditions, respectively, as shown in our previous work [37].

### Bacteriophage isolation and identification

The 12 bacterial species described in the preceding section were isolated from the larval gut and used as hosts to screen phages. The phages were isolated from the housefly larval gut and the Tai'an Sewage Treatment Plant as described previously [35, 37, 38].

Among the 12 bacterial phages, five were considered to be beneficial bacterial phages, including EhX phage Phc, KX phage Pkc, Ec phage Pec, Lf phage Pfc and Bs phage Pbc, and two were considered to be harmful bacterial phages, including Y12 phage Ppc and Ef phage EfP.

### Oral administration of bacteria/phage cocktail into the larvae

We studied the effects on housefly larval growth of a beneficial bacteria cocktail consisting of the beneficial bacteria EhX, KX, Ab, Ec, Lf and Bs, a harmful bacteria cocktail consisting of the harmful bacteria Y12 and Ef, a beneficial bacteria phage cocktail consisting of EhX phage Phc, KX phage Pkc, Ec phage Pec, Lf phage Pfc and Bs phage Pbc and a harmful bacteria phage cocktail consisting of Y12 phage Ppc and Ef phage EfP.

First, 10 uniformly sized 1-day-old larvae showing normal breeding behavior and good growth were each added to a separate centrifuge tube. Each group was repeated three times, and a piece of gauze was placed between the tube and the lid to prevent the larvae from escaping. The housefly larvae from different groups were fed with Luria-Bertani liquid medium (LB) (negative control) and the following cocktails composed of: (i) five beneficial bacterial phage (BBP); (ii) two harmful bacterial phage (HBP); (iii) five beneficial bacteria (BB) and (iv) two harmful bacteria (HB). Each cocktail contained 10^9^ plaque-forming unit (PFU)/ml or 10^9^ colony-forming unit (CFU)/ml of each phage or bacteria, respectively, mixed at a 1:1 ratio based on our previous research [37].

Beginning on the first day of larval rearing, four larvae were simultaneously removed from each tube every day to record their length and weight, and to observe biological indicators, including pupation rate, emergence rate and developmental duration in each tube.

### Housefly larvae crawling ability assay and trypan blue staining

The crawling ability of housefly larvae was assessed in an assay involving trypan blue staining using the protocol described previously [29].

### Plate confrontation assay between beneficial and harmful bacteria in the housefly larval gut

We inoculated Ef cultures on half of the nutrient agar plate; the opposite side was used as the negative control. We then added 10 µl of gut bacteria, including EhX and Ec, to the filter papers according to our previously described protocol [37].

### Effects of feeding bacteria/phage cocktail on housefly larval phenoloxidase activity

Larval phenoloxidase activity was determined using the method described previously [28, 29].

### Determination of growth rate of culturable and phage-resistant bacteria in the housefly larval gut

To determine the predominant bacterial species in the niche, we analyzed the growth rate of culturable and phage-resistant bacteria. Bacterial growth was evaluated using nutrient agar plates under aerobic conditions, following which six beneficial bacteria (EhX, KX, Ec, Lf, Bf, and Ab), five harmful bacteria (Y12, Ef, Sm, Ps, and Pv) and one neutral bacteria (NB) (Ll) were inoculated into LB liquid medium and cultured at 37 °C overnight (OD_600_ > 1). Two 6-mm–wide sterile filter papers were placed symmetrically on both sides of the agar medium, and 10 µl of EhX was added to the filter papers.

The phage-resistant bacteria R1 and R4 were obtained after coculturing the phages with the host bacteria for 1 and 4 days, respectively. In the case of R1, the harmful phage-resistant bacteria (RHB) (RY12: resistant Y12; REf: resistant Ef) and beneficial phage-resistant bacteria (RBB) (REhX: resistant EhX; RKX: resistant KX; REc: resistant Ec; RLf: resistant Lf; RBs: resistant Bs) were inoculated in LB liquid medium and cultured at 37 °C overnight (OD_600_ > 1). Then, 10 µl of REhX was added to the filter papers. All plates were cultured at 37 °C under aerobic conditions for different periods. Finally, the diameter of the bacterial colony was recorded to evaluate their growth rate. The experiments were conducted with six independent biological replications.

### Sequencing and bioinformatics analysis

Whole-genome sequencing of the phages using the Illumina HiSeq 4000 platform (Illumina Inc., San Diego, CA, USA) and the phage sequences were de novo assembled by the MetaviralSPAdes tool according to our previous research [37]. The phage genomes used in this study are available in GeneBank under accession numbers OQ884026 (Pkc), OQ884028 (Pec), OQ884027 (Ppc), OQ884030 (Pbc), MZ669808 (Phc) [35], OQ884029 (Pfc) and OP889240 (EfP).

We performed 16S rRNA gene high-throughput sequencing of the housefly gut microbiota and bioinformatics analysis based on our previous protocols with slight modifications [38]. The hypervariable V3-V4 region of the bacterial 16S rRNA gene was amplified with the primers 341F and 805R. According to the recommended parameters of the quantitative insights into microbial ecology 2 divisive amplicon denoising algorithm 2, the primers were removed, followed by denoising, merging and removal of chimera by the dada2 denoise-paired function to obtain the representative sequences (amplicon sequence variants [ASV]) and ASV table. The taxonomy of ASVs was then assigned by classify-sklearn (QIIME2 plugin feature-classifier) against the SILVA database (SILVA_138.1) [39, 40]. The alpha and beta diversity were computed using the QIIME 2 command “qiime diversity core-metrics-phylogenetic.” We used Phylogenetic Investigation of Communities by Reconstruction of Unobserved State (PICRUSt) software and Kyoto Encyclopedia of Genes and Genomes pathway functions for functional annotation of the 16S rRNA-based metagenome.

### Metabolite extraction and liquid chromatography-tandem mass spectrometry

Following the protocol established in our previous research [37], the larval body surface was first thoroughly disinfected with 75% alcohol and then the samples were frozen in liquid nitrogen and stored at − 80 °C for subsequent untargeted metabolomics. Each group was studied in triplicate (3*n* = 3 × 5).

Untargeted metabolomics of the larval samples by liquid chromatography-tandem mass spectrometry (LC–MS/MS) was performed by the KeGene Science & Technology Co. Ltd. (Shandong, China) using a Vanquish Ultra-High-Performance Liquid Chromatography system (Thermo Fisher Scientific, Waltham, MA, USA) coupled with an Orbitrap Exploris™ 480 mass spectrometer (Thermo Fisher Scientific). The raw data were collected separately in the positive and negative ion modes and was converted to mzXML format by ProteoWizard (version 3.0.6150). Then, peak identification and retention time alignment for all mzXML data were performed by the XCMS (version 1.46.0) package. Finally, the metabolites were identified by metDNA (http://metdna.zhulab.cn/). Using the VIP values of the first two main components of the partial least squares-discriminant analysis (PLS-DA) model, combined with the differential fold change obtained by univariate analysis and Student’s t-test results, we screened differential metabolites according to our previous research [41].

### Statistical analysis

The experimental data were analyzed by Microsoft Excel 2021 (Microsoft Corp., Redmond, WA, USA and IBM SPSS Statistics 20 statistical software (SPSS IBM, Armonk, NY, USA. All data are expressed as the mean ± standard deviation (SD). The effects of different treatments on the body weight and length of housefly larvae were compared using two-way analysis of variance. Significance analysis was performed by Sidak’s multiple comparisons test (*P* < 0.05).

## Results

### Isolation and identification of gut bacteria from housefly larvae

**Fig. 1:**
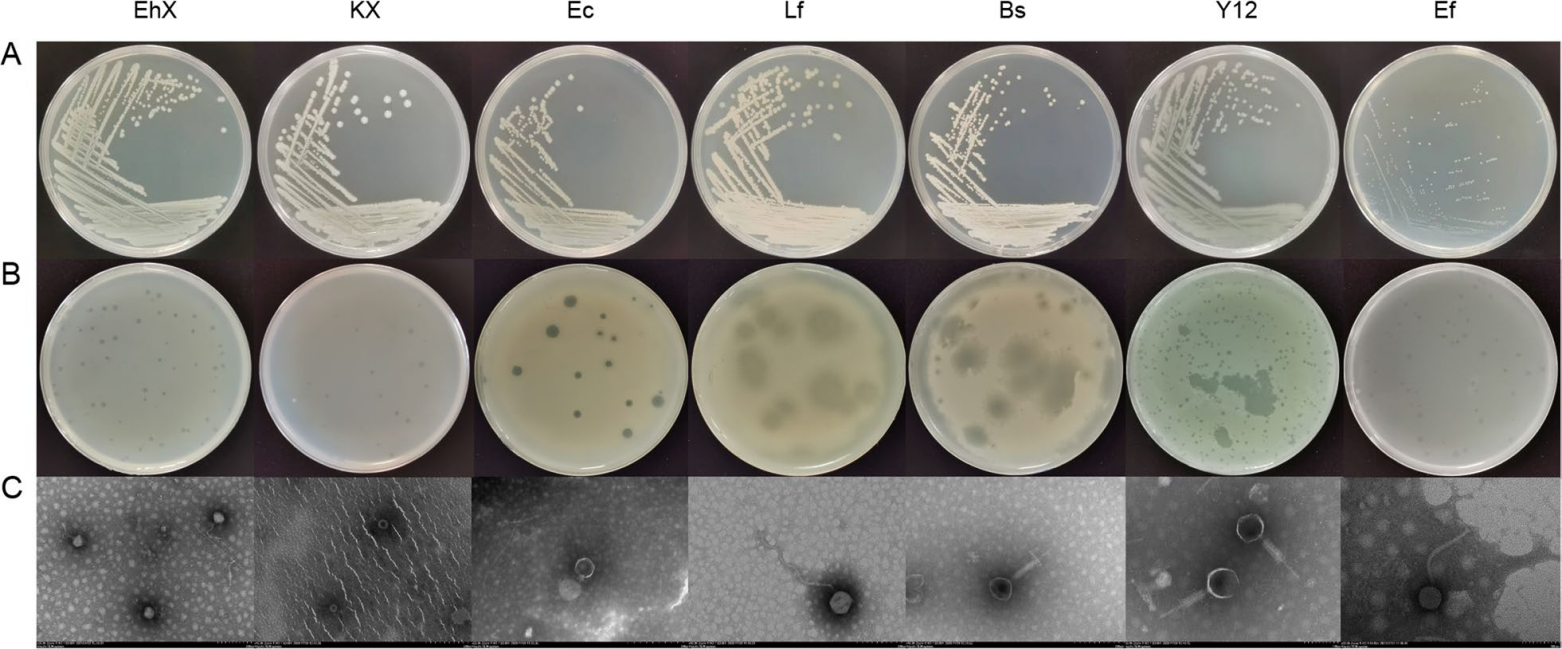
**a** Colony morphology of seven kinds of bacteria. **b** Morphology of 7 kinds of phages. **c** Electron micrograph of 7 kinds of phages. EhX, *Enterobacter hormaechei*; KX, *Klebsiella pneumoniae*; Ec, *Enterobacter cloacae*; Lf, *Lysinibacillus fusiformis* Bs: *Bacillus safensis*; Y12, *Pseudomonas aeruginosa*; Ef, *Enterococcus faecalis*

In a previous study we isolated 10 culturable bacteria, including EhX*,* KX*,* Y12*,* Ab*,* Ps*,* Ec*,* Ll*,* Lf*,* Pv, and Bs [28]. We also isolated and identified bacteria from the larval intestine [36]. Sm and Ef were isolated from larval intestines under aerobic and anaerobic conditions, respectively (Fig. 1A).

### Isolation of bacteriophage from the housefly larval gut or sewage

We isolated six phages from the larval gut, namely EhX phage Phc, KX phage Pkc, Y12 phage Ppc, phage Pec, Lf phage Pfc and Bs phage Pbc; in contrast, Ef phage EfP was isolated from sewage. After purifying the isolated phages, uniform plaques were cultured on the double-layer plates (Fig. 1b). Transmission electron microscope analysis revealed that the phages Pkc and Pec probably belong to family* Autographiviridae*, Ppc to family* Myoviridae*, Pbc to family* Herelleviridae*, Phc to family* Drexlerviridae* [35] and Pfc and EfP to family* Siphoviridae* (Fig. 1c). Genome sequencing analysis showed that the total length of the Phc, Pkc, Ppc, Pec, Pfc, Pbc and Efp genomes were 52,494, 42,833, 65,696, 44,357, 41,198, 151,475 and 41,871 bp, respectively (Additional file 1: Figure S1).

### Effects of bacteria/phage cocktails on housefly larval growth and crawling ability

**Fig. 2:**
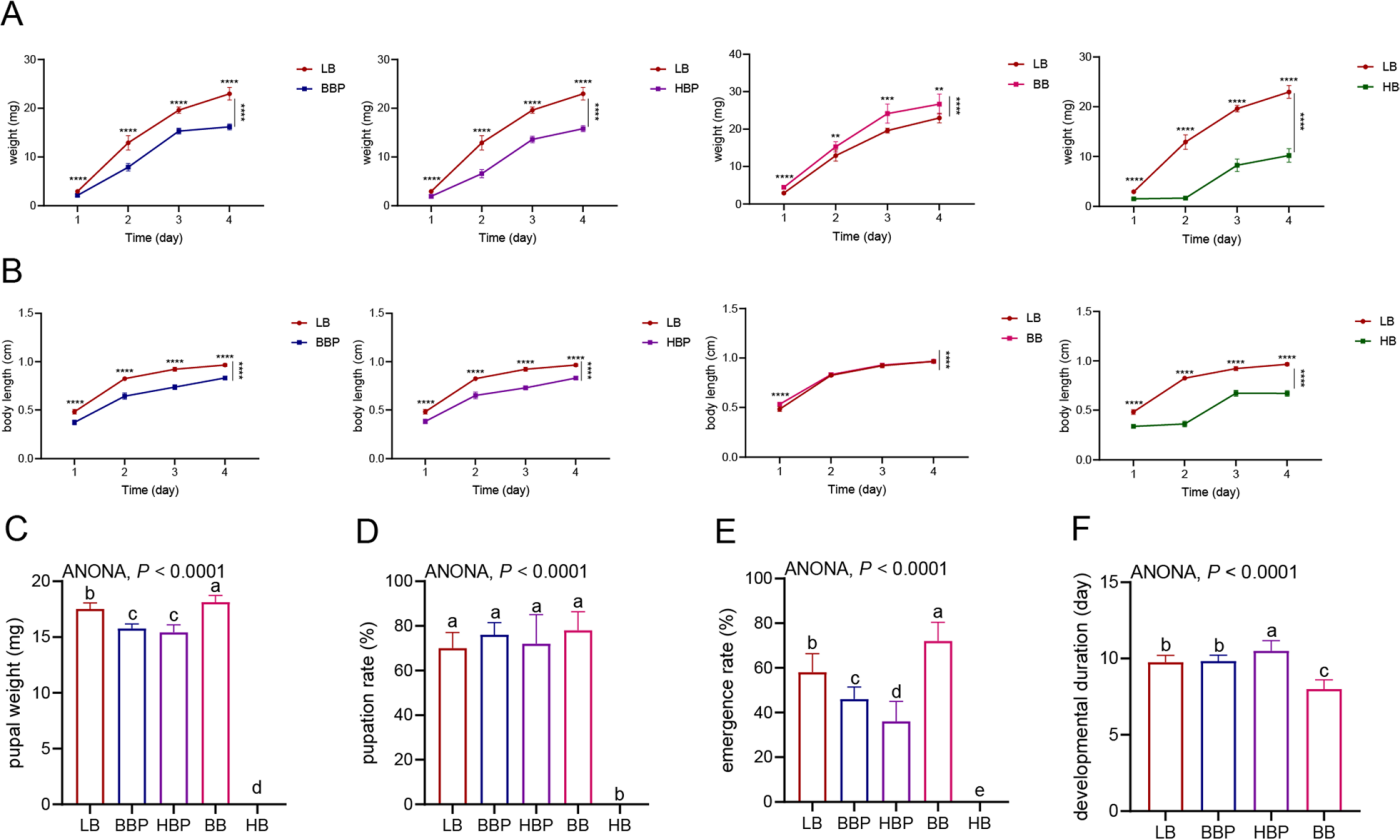
Effects of different treatments on the growth and development of housefly larvae. The different treatments had significant effects on the body weight (**a**), body length (**b**), pupal weight (**c**), pupation rate (**d**), eclosion rate (**e**) and development duration (**f**) of housefly larvae. The BB cocktail consisted of 5 beneficial bacteria phage (EhX phage Phc, KX phage Pkc, Ec phage Pec, Lf phage Pfc and Bs phage Pbc) mixed at 1:1 at the same concentration of 10^9^ PFU/ml. The HBP cocktail consisted of 2 harmful bacteria phage (Y12 phage Ppc and Ef phage EfP) mixed at 1:1 at the same concentration of 10^9^ PFU/ml. The BB cocktail consisted of 5 beneficial bacteria (EhX, KX, Ab, Ec, Lf and Bs) were mixed at 1:1 at the same concentration of 10^9^ CFU/ml. The HB cocktail consisted of 2 harmful bacteria (Y12 and Ef) mixed at 1:1 at the same concentration of 10^9^ CFU/ml. BB, Beneficial bacteria cocktail; BBP, beneficial bacteria phage cocktail; CFU, colony-forming units; HB, harmful bacteria cocktail; HBP, harmful bacteria phage cocktail; LB, Luria-Bertani liquid medium (negative control); PFU, plaque-forming units

To analyze the effects of the bacteria/phage cocktail on the larvae, we added these cocktails to the basal diet of the larvae and monitored larval growth using specific biological indicators, including body weight, body length, pupation rate, emergence rate and developmental duration. Among the 12 isolated bacteria, the BB group consisted of EhX, KX, Ab, Ec, Lf and Bs; the HB group included Y12 and Ef; and the BBP group contained EhX phage Phc, KX phage Pkc, Ec phage Pec, Lf phage Pfc and Bs phage Pbc. Y12 phage Ppc and Ef phage EfP were included in the HBP group. Our results revealed that while larval growth was promoted in the BB group, the opposite was observed in the other groups (Fig. 2). These results show that oral administration of various beneficial bacteria enhanced larval growth, while oral administration of the harmful bacteria/phage cocktails inhibited larval growth by suppressing various beneficial bacteria.

**Fig. 3:**
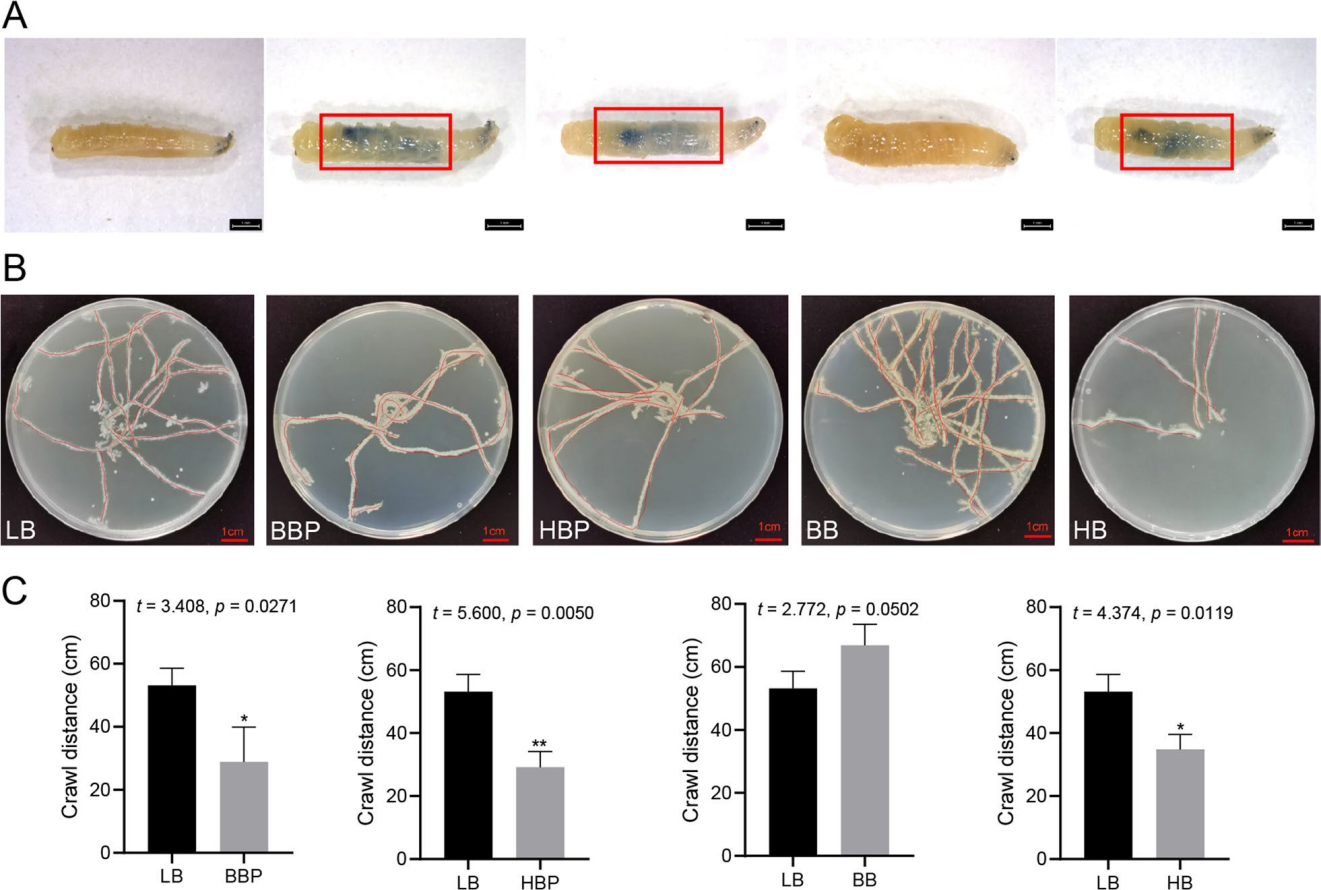
**a** Intestinal damage of housefly larvae. The larvae in the BBP group, HBP group and HB group showed a blue color due to intestinal damage (red frame). **b** Crawl trace of housefly larvae on the crawl trace culture medium. Values are the mean ± SD of triplicate treatments. Asterisks indicate significant differences at *P < 0.05 and ** P < 0.01. BB, Beneficial bacteria cocktail; BBP, beneficial bacteria phage cocktail; HB, harmful bacteria cocktail; HBP, harmful bacteria phage cocktail; SD, standard deviation

The crawling trail results showed that both the crawling ability and crawl distance were significantly decreased in larvae in the BBP, HBP and HB groups (Fig. 3b, c), but not in those in the BB group (Fig. 3b, c).

### Effects of the bacteria/phage cocktail on housefly larval intestinal tissues

We performed histological analysis in an attempt to understand the mechanism underlying the effect of the bacteria/phage cocktails on larval development. Compared with the trypan blue-stained images of the larval gut in the BB and LB groups, the stained images of the larval gut of the BBP, HBP and HB groups showed a clear blue intestinal tract. These results indicated that the latter three treatments directly affected larval growth by damaging their intestinal tissues (Fig. 3a).

### Effects of the bacteria/phage cocktail on housefly larval intestinal microflora

**Fig. 4:**
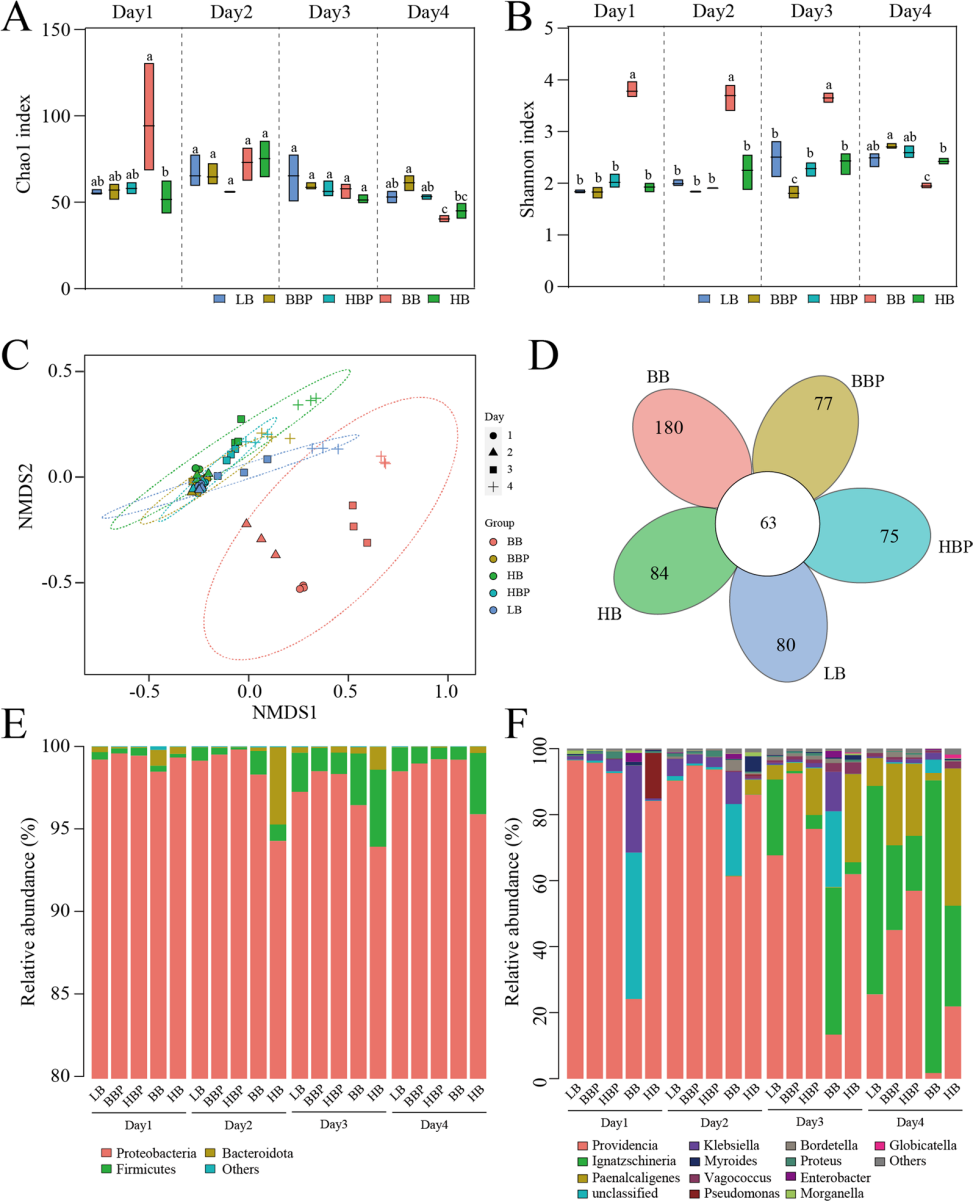
Bacterial richness and diversity of samples. **a** Chao1 index. **b** Shannon index. The data were compared by one-way analysis of variance. The Brown-Forsythe test was used for significance analysis. Values are the mean ± standard deviation from of triplicate treatments. **c** NMDS of bacterial community structure in 7 groups. Each symbol represents a sample of intestinal bacteria. **d** Flower plot showing the unique and shared OTUs of the intestinal bacteria in housefly larval samples. **e** Relative abundances of the top 3 phyla in housefly larval samples. **f** Relative abundances and distributions of the top 12 genera in housefly larval samples. The dominant bacterial genera based on OTUs identified in samples from each housefly larvae are shown. NMDS, Non-metric multi-dimensional scaling; OTU, operational taxonomic unit; SD, standard deviation

We performed 16S rRNA gene sequencing of the intestinal bacteria from the different larval groups to study the effect of the changes in various bacteria on the composition of the bacterial community. The pipeline started with 6,387,140 single reads and yielded 4,781,413 nonchimeric sequences, which corresponded to an average recovery of 79,690 sequences or 74.93% per sample (*n* = 60). After filtering using QIIME2 with the DADA2 algorithm, we obtained 665 ASVs, of which 202 were present in more than one sample. Therefore, wer concluded that oral administration of bacteria/phage cocktails affected the composition of the bacterial community (Fig. 4). The Chao1 and Shannon indices showed that the bacterial diversity was altered in the different groups (Fig. 4a, b). Non-metric multi-dimensional scaling showed significant differences in the larval intestinal microbial communities between the BB group and other groups (Fig. 4c). Therefore, we concluded that changes in the beneficial or harmful bacteria communities significantly altered the larval gut microbiota in the different groups, as shown in the flower plot (Fig. 4d).

The analysis of dominant phyla and genera showed that larval samples in the different treatment groups had different bacterial community structures at the genus level, with only slight differences at the phylum level (Fig. 4e, f). The composition of the 12 most abundant larval intestinal bacterial genera was significantly altered in the different treatment groups. Compared with the control group (LB), the proportion of beneficial bacteria, such as *Klebsiella* (1d, 2d, 3d and 4d) and *Enterobacter* (1d, 2d, 3d and 4d), increased and the proportion of harmful bacteria, such as *Providencia* (1d, 2d, 3d and 4d), *Morganella* (1d and 4d) and *Pseudomonas* (1d), decreased in the BB group (Fig. 4e, f). However, compared with the control group (LB), the proportion of harmful bacteria, such as *Pseudomonas* (1d, 2d, and 3d in the HB group and 1d in the HBP group), *Providencia* (2d, 3d, and 4d in the BBP and HBP groups) and *Morganella* (3d and 4d in the HB group and 2d and 3d in the BBP and HBP groups) increased. The beneficial bacteria, such as *Klebsiella* (1d, 2d, 3d, and 4d in the HB group and 2d in the BBP and HBP groups) and *Enterobacter* (1d and 2d in the HB group and 2d in the BBP and HBP groups) decreased. We assume that the oral administration of harmful bacteria bacteriophage cocktails had the most pronounced effect on the larval gut microbiota, facilitating the growth of harmful bacteria and inhibiting larval growth. Therefore, we speculate that the change in the proportion of dominant bacteria in the larval gut is another key factor that affects the housefly’s health.

To determine the interaction between culturable and beneficial/harmful bacteria, we performed antagonism assays. Previous experiments showed that beneficial bacteria can inhibit the growth of harmful bacteria [42] and vice versa [36]. The antagonism assay showed that the growth of EhX and Ec was inhibited by Ef, consistent with our previous research (Additional file 2: Figure S2).

### Effect of different bacteria/phage cocktails on the gut microbiota network

**Fig. 5:**
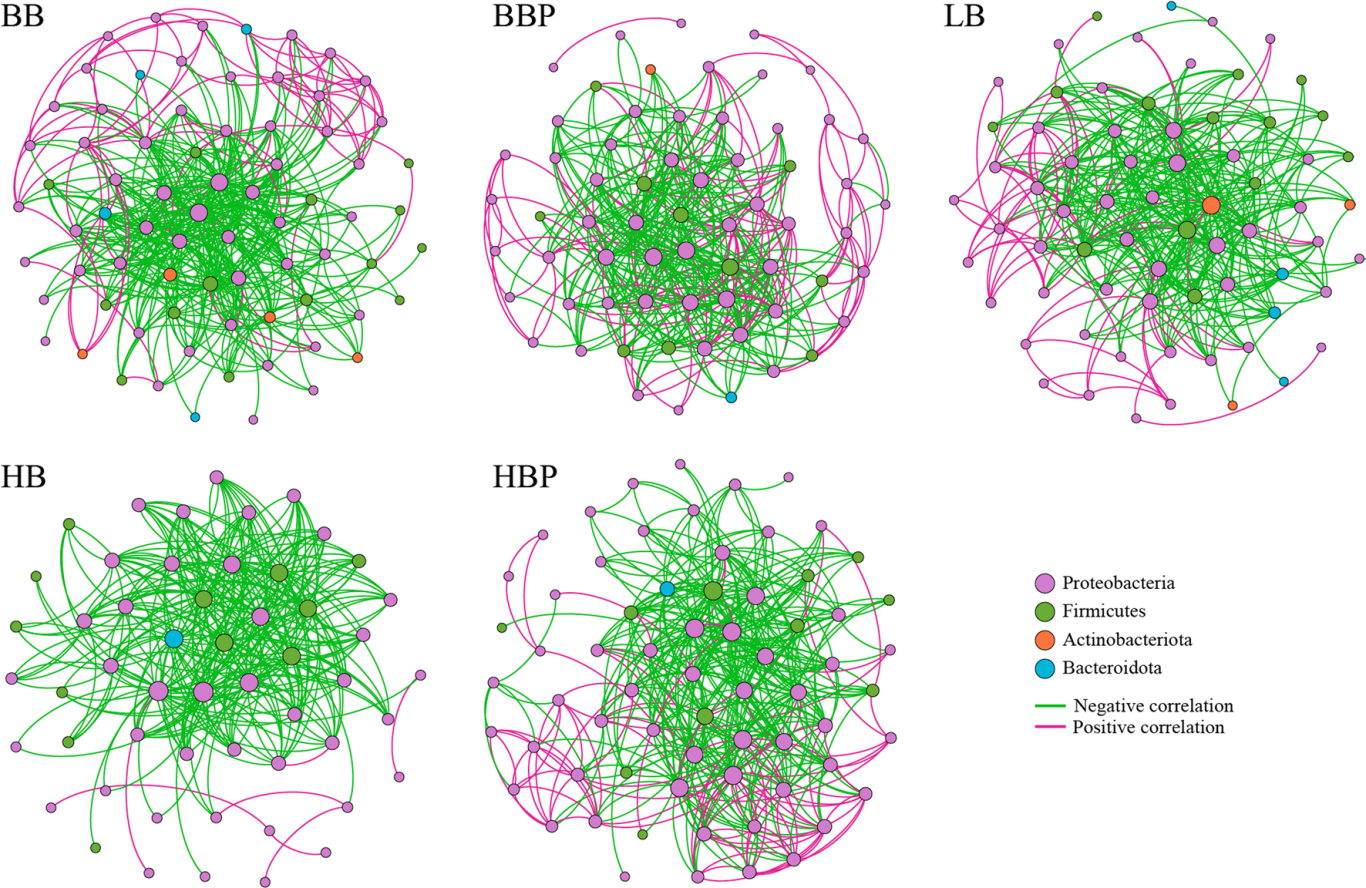
Intestinal bacterial co-occurrence microbiome networks between different groups. Each point in the graph represents a species, and those related species are connected by a line. Red lines represent positive correlations, and green lines represent negative correlations. The node colors represent the taxon classifications at the phylum level

To analyze the bacterial correlations within the bacterial community, we constructed a network based on the Spearman correlation. In all the samples, we observed a high degree of connectivity between Proteobacteria (Fig. 5). Compared with the LB group, the interaction between Proteobacteria (81.25%, 82.09%, 70.27%, and 75.47%) was significantly enhanced in the BBP, HBP, BB and HB groups. This might be due to increased association between some harmful bacteria in Proteobacteria in the BBP, HBP and HB groups, while that between some beneficial bacteria in Proteobacteria increased in the BB group.

### Effects of feeding bacteria/phage cocktail on housefly larval phenoloxidase activity

**Fig. 6:**
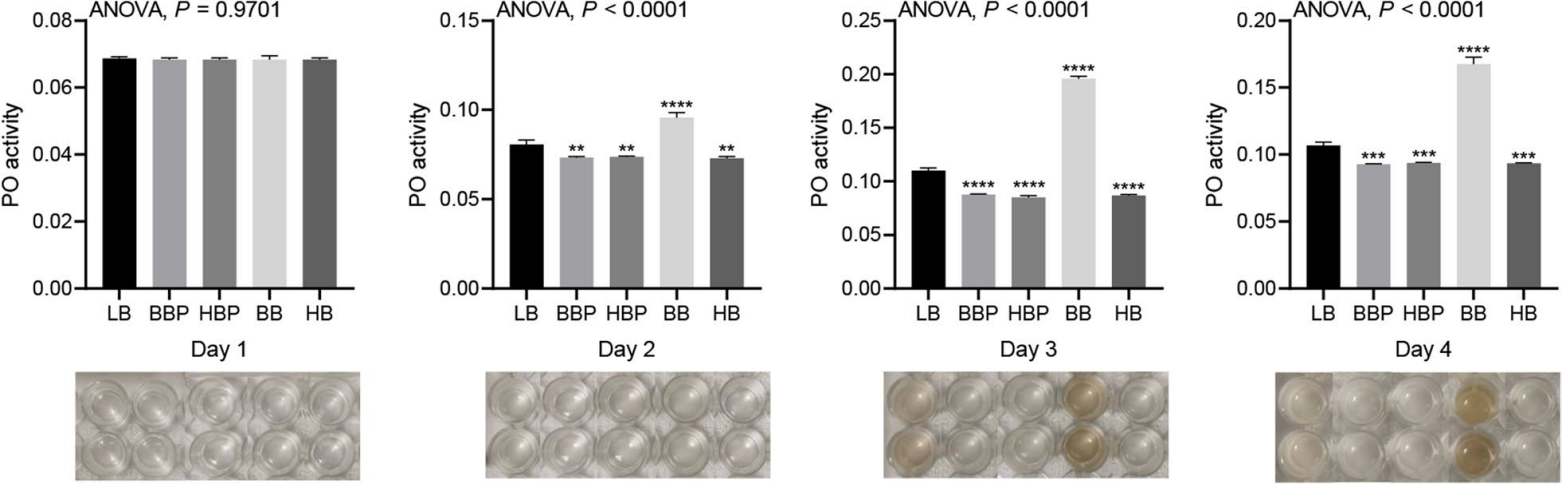
Changes in the phenoloxidase activity in the hemolymph of housefly larvae on days 1–4 post cocktail administration in the different groups. Data are shown as the mean ± standard error of the mean. The data were compared by one-way analysis of variance. Asterisks indicate significant differences at *P < 0.05, **P < 0.01, ***P < 0.001 and **** P < 0.0001

We studied the effects of the bacteria/phage cocktail on phenoloxidase activity in the larvae hemolymph to understand the larval immune response. The phenoloxidase activity in the hemolymph was significantly increased in the BB group but decreased in the other groups on days 2, 3 and 4 days post cocktail administration (Fig. 6).

### Analysis of the growth rate of culturable and phage-resistant bacteria in housefly larval intestines

**Fig. 7:**
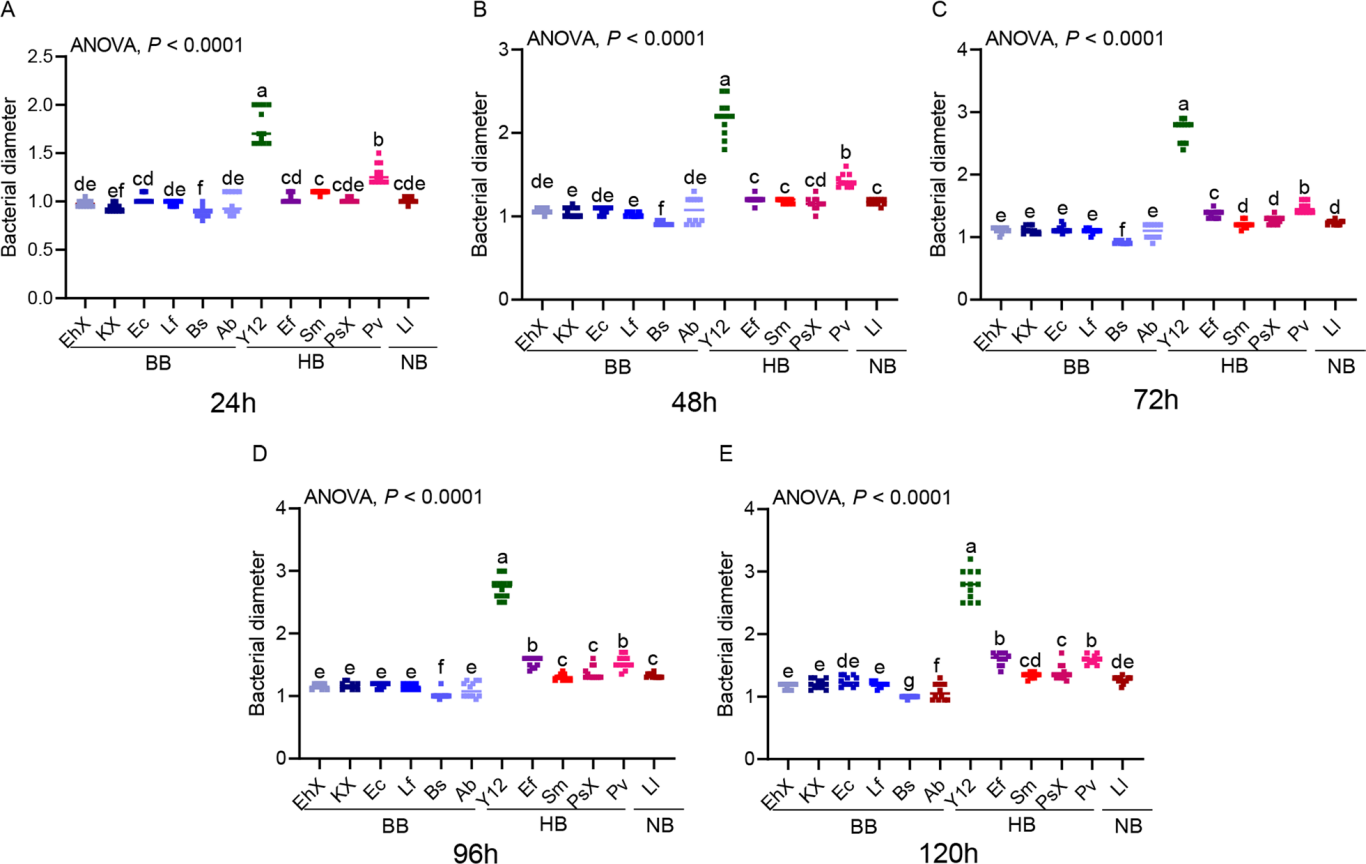
Growth rate of culturable bacteria in gut of housefly larvae. To determine the growth rate of culturable bacteria in the gut of housefly larvae, bacterial growth tests were carried out in NA medium plates under aerobic culture conditions. BB (EhX, KX,Ec, Lf, Bf and Ab), HB ( Y12, Ef, Sm, PsX and Pv) and NB (Ll) were inoculated in LB liquid medium and cultured at 37 °C overnight (OD_600_ > 1.0). Two 6 mm-diameter sterile filter papers were placed symmetrically on both sides of the agar medium, and 10 µl EhX was added to the filter papers. All plates were cultured at 37 °C under the aerobic condition for 24 (**A**), 48 (**B**), 72 (**C**), 96 (**D**) and 120 h (**E**), respectively. The growth diameter of bacteria was recorded. Other bacteria were cultured and the growth diameter measured similarly. The growth rate was evaluated by measuring the colony size. The experiments were conducted with three independent biological replications. The data were compared by one-way analysis of variance. BB, Beneficial bacteria cocktail; BBP, beneficial bacteria phage cocktail; HB, harmful bacteria cocktail; HBP, harmful bacteria phage cocktail; LB, Luria-Bertani (negative control); NA, neutral agar; NB, neutral bacteria

To determine the predominant bacterial species in the niche, we analyzed the growth rates of culturable bacteria and phage-resistant bacteria. The results revealed that all harmful bacteria grew rapidly (except Ps after 24 h) (Fig. 7) and easily colonized and increased in the BBP group, hindering larval development.

**Fig. 8:**
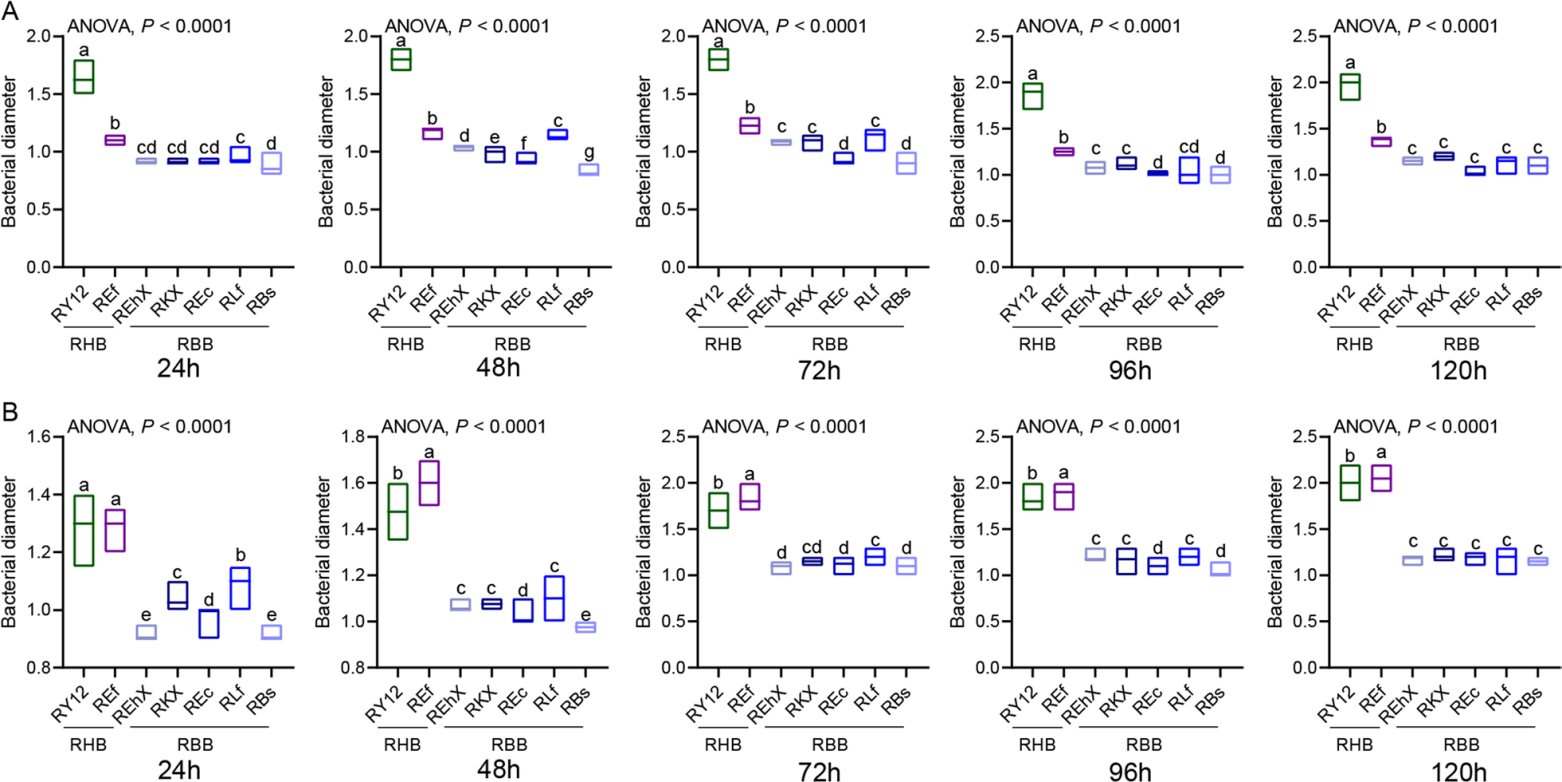
Determination of growth rate of phage-resistant bacteria in culturable bacteria targeted by phages in the gut of housefly larvae. To determine the growth rate of phage-resistant bacteria of culturable bacteria targeted by phages in the gut of housefly larvae, phage-resistant bacteria R1 and phage-resistant bacteria R4 were obtained after co-culture of phage and host bacteria for 1 (**A**) and 4 days (**B**), respectively. Bacterial growth tests were carried out in NA medium plates under aerobic culture conditions. In the case of phage-resistant R1 bacteria R1, RHB (resistant Y12 [RY12], resistant Ef [REf]) and RBB (resistant EhX [REhX], resistant KX [RKX],resistant Ec [REc], resistant Lf [RLf], resistant Bs [RBs]) were inoculated in LB liquid medium and cultured at 37 °C overnight (OD_600_ > 1.0). Two 6 mm-diameter sterile filter papers were placed symmetrically on both sides of the agar medium, and 10 µl REhX: resistant EhX was added to the filter papers. All plates were cultured at 37 °C and cultured under aerobic conditions for 24, 48, 72, 96 and 120 h, respectively. The growth diameter of bacteria was recorded. Other bacteria were cultured and the growth diameter measured similarly. The growth rate was evaluated by measuring the colony size. The experiments were conducted with three independent biological replications. The data were compared by one-way analysis of variance. LB, Luria-Bertani (negative control); NA, neutral agar; RBB, phage-resistant bacteria of beneficial bacteria; RHB, phage-resistant bacteria of harmful bacteria

The phage-resistant bacteria R1 and R4 were obtained after co-culturing the phages with the host bacteria for 1 and 4 days, respectively. The results revealed that phage-resistant harmful bacteria (RY12: Y12; REf: Ef [except R1]) grew the fastest (Fig. 8). This observation is consistent with the findings showing an increased abundance of harmful bacteria, such as *P. aeruginosa* in the HBP group. Due to their higher proliferation ability, these harmful bacteria inhibited larval growth.

### Metabolic alterations related the housefly larvae gut microbiota dysbiosis

**Fig. 9:**
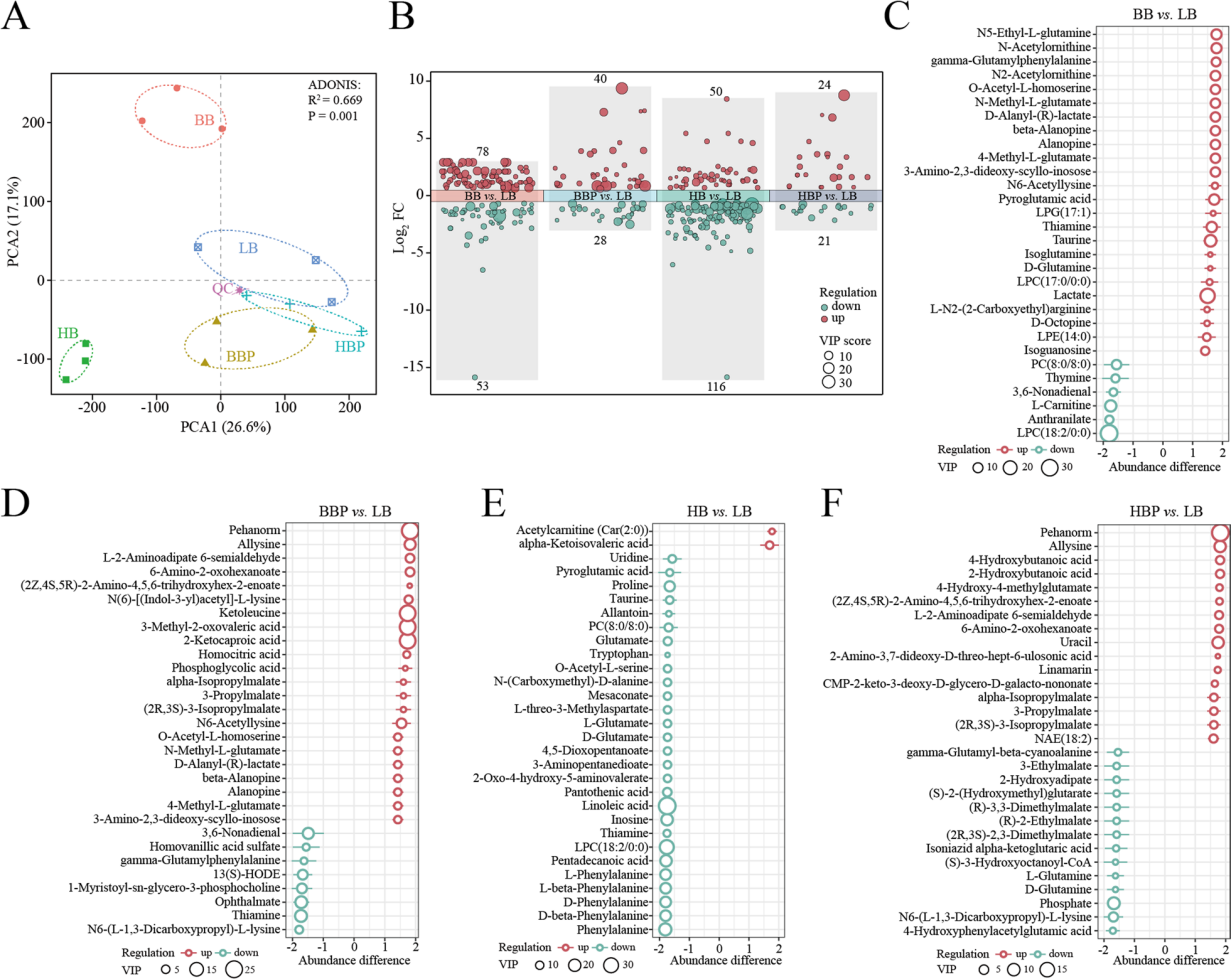
Impact of different treatment groups on the metabolic profile of housefly larvae. **a** Principal component analysis plots of the metabolite composition of samples from different treatment group. Each symbol represents a sample, with different symbol shapes denoting different groups. The circular line indicates the 95% confidence interval. **b** Metabolite changes between different treatment groups. Fold change of A versus B is calculated as A/B, ‘up’ represents VIP > 1, FC > 1.5, *P* < 0.05, ‘down’ represents VIP > 1, FC < 0.667, *P* < 0.05. Volcano plot for the expression pattern of each metabolite. Red and blue points represent upregulated metabolites and downregulated metabolites, respectively. The discriminatory metabolites were identified and ranked according to the extent of the difference by comparing the BB group versus the LB group (**c**), the BBP group versus the LB group (**d**), the HB group versus the LB group (**e**) and the HBP group versus the LB group (**F**). Green and red circles denote BB, BBP, HB or HBP downregulated and BB, BBP, HB or HBP upregulated metabolites, respectively. BB, Beneficial bacteria cocktail; BBP, beneficial bacteria phage cocktail; FC, fold change; HB, harmful bacteria cocktail; HBP, harmful bacteria phage cocktail; LB, Luria-Bertani (negative control) VIP, variable importance in projection

To study the alterations in the metabolic patterns correlated with the larval gut microbiota dysbiosis, we evaluated the metabolomes of the different groups using LC–MS/MS and identified a total of 1063 metabolites. Principal component analysis showed that the metabolites were separated between different groups and were clustered within each group (*R*^2^ = 0.669, *P* = 0.001, Adonis test) (Fig. 9a). Of these, 131, 68, 166 and 45 metabolites were significantly different in the pairwise comparisons BB versus LB, BBP versus LB, HB versus LB and HBP vesus LB, respectively (Fig. 9b). Previous studies indicated that multivariate variables affecting variable importance in projection (VIP) values facilitate distinguishing between different treatment groups as a selection of metabolites is crucial for more accurate classification. In this study, we selected the top 30 significant metabolites with VIP > 1 (Fig. 9c–f). We found that the metabolites between BB versus LB, BBP versus LB, HB versus LB and HBP versus LB were significantly different. Among the differential metabolites, six were downregulated and 24 were up-regulated in the BB group, and eight were downregulated and 22 were up-regulated in the BBP group (Fig. 9c, d). Also, 28 were downregulated and two were upregulated in the HB group, and 14 were downregulated and 16 were upregulated in the HBP group (Fig. 9e, f). In addition, the composition of the metabolites was affected as some beneficial metabolites, such as lactate, taurine, and D-glutamine, were upregulated in the BB group, which promoted larval development (Fig. 9c). However, beneficial metabolites, such as linoleic acid, taurine, D- and L-glutamine, were downregulated in the BBP, HBP and HB groups, inhibiting larval development (Fig. 9d–f). These changes in the larval differential metabolites might also affect larval health. We further explored the correlation between altered gut microbiota and larval metabolites (Additional file 3: Figure S3). Among the 12 genera, we observed significant correlations among different metabolites in the BB versus LB, BBP versus LB, HB versus. LB and HBP versus LB groups, which also significantly correlated with the gut microbiota alterations in these groups. Interestingly, when measuring the correlation between differential metabolites and the top 12 genera, we found that some beneficial metabolites, such as lactate, taurine, D- and L-glutamine, were positively correlated with beneficial bacteria, such as *Klebsiella* and *Enterobacter*, and negatively correlated with harmful bacteria, such as *Providencia* and *Morganella*. This further emphasized the significance of these bacteria. Combined with the correlation between altered gut microbiota and larval metabolites, we speculated that the shift in the gut microbiota contributed to the metabolite changes, which, in turn, affected larval development.

## Discussion

Our findings showed increased larval phenoloxidase activity and enhanced larval growth in the BB group. However, the larval intestinal community structure and larval growth were negatively affected in the other groups. Furthermore, the larval phenoloxidase activity was decreased in the BBP, HBP and HB groups. Metabolic analysis shows that, despite the disturbance to the gut microbial community, oral administration of a beneficial bacteria cocktail further affected the intestinal metabolites. We assume that the disrupted gut flora induced metabolic alterations in different groups. The larval gut microbiota is affected differently, which further induced intestinal metabolic alteration in the BBP, HBP and HB groups. The downregulation of some beneficial metabolites, such as linoleic acid, taurine, d- and l-glutamine, inhibited larval development in the BBP, HBP and HB groups.

Previously, *E. hormaechei* was shown to accelerate housefly larval growth by inhibiting some pathogenic strains and modulating the gut flora [42]. Phage-targeted knockdown of *E. hormaechei* disturbed the gut microbiota and affected the insect’s health [35]. However, the effects of the changes in various beneficial/harmful bacteria on housefly larval growth are unclear.

We observed that increased abundance of beneficial bacteria, including *Klebsiella* and *Enterobacter* in the BB group, concurrently with decreased abundance of harmful bacteria, including *Providencia*, *Morganella* and *Pseudomonas*. In comparison, beneficial bacteria, such as *Klebsiella*, and *Enterobacter*, were less abundant in the BBP, HBP and HB groups, while harmful bacteria, such as *Providencia* (BBP and HBP), *Pseudomonas* (HBP and HB) and *Morganella* (BBP, HBP, and HB) were more abundant. Multi-resistant *Klebsiella pneumoniae* strains in the gut of the black soldier fly *Hermetia illucens* helped its larvae survive against sulfonamides (SAs) and cadmium (Cd) stress [43]. Under aseptic rearing, inoculation with *E. cloacae* increased pupal weights and male fitness of the transgenic diamondback moth [44]. Given these effects of *Klebsiella* and *Enterobacter*, we assumed that they might positively influence the early development of the housefly larval.

The authors of some previous studies reported that some pathogenic bacteria, such as *Pseudomonas*, *Providencia*, *Morganella* and *Serratia* adversely affected other insects. *P. aeruginosa* is pathogenic toward *Drosophila*, whiteflies and nematode larvae [45]. It also causes major changes in the housefly larval intestinal microbiota, leading to larval death [36]. *Providencia* spp. are also known to inhibit insect development, although the death rate and host immune response are different in *Drosophila melanogaster* infected with *Providencia sneebia* and *P. rettgeri* [46]. The abundance of beneficial microbes was higher in virgin *Spodoptera frugiperda* females while the abundance of pathogens, such as *Morganella* spp, and *Serratia* spp., was higher in the females mated with multiple males [47].

Our previous research shows that beneficial bacteria, including *E. hormaechei* [42], *E. cloacae* and *K. pneumoniae*, inhibit the growth of harmful bacteria, such as *P. aeruginosa*, *P. stuartii* and *P. vermicola*. However, harmful bacteria, including *P. aeruginosa*, can inhibit the growth of beneficial bacteria, such as *E. hormaechei*, *K. pneumoniae* and *E. cloacae* [36]. The plate confrontation assay showed that *E. faecalis* Ef inhibited the growth of EhX and Ec. Based on the results of our plate antagonism experiment, we assume that some beneficial bacteria might compete with other strains for nutrition and inhibit the growth of harmful bacteria, promoting larval growth. Similarly, some harmful bacteria inhibit the growth of beneficial bacteria, thereby inhibiting larval growth.

Interference with the abundance and composition of bacteria in the larval gut after oral administration of different microbial combinations affected the larval growth. The beneficial gut bacteria, such as *Klebsiella* and *Enterobacter*, increased in the BB group, improving larval development, whereas an increased abundance of harmful bacteria, such as *Pseudomonas* and *Morganella*, in the gut in the HB group inhibited larval development. Similarly, an increased abundance of harmful bacteria, such as *Providencia* and *Pseudomonas,* inhibited larval growth in the BBP and HBP groups. The abundance of harmful bacteria, such as *Providencia*, increased significantly within a short period in the BBP group, probably due to the knockdown of beneficial bacteria by their targeting phages, resulting in the proliferation of harmful bacteria. Moreover, due to the rapid growth rate of harmful bacteria, they can easily colonize and hinder larval growth. The abundance of harmful bacteria, such as *P. aeruginosa,* increased in the HBP group. Our previous results revealed that specific bacteria can be knocked down by its targeting phage [29, 35], enabling gut microbial regulation. However, the abundance of harmful bacteria was not significantly reduced in the HBP group. We speculate that as the effect of oral administration of a single bacteriophage is less potent than that of a bacteriophage cocktail on the larval gut, in this case harmful bacteria were enhanced and larval growth inhibited. Therefore, the bacterial composition in the larval gut was imbalanced, significantly affecting larval growth.

Previous studies have demonstrated that insect growth, survival and fecundity can be cooperatively regulated by metabolism [34, 48, 49]. Oral administration of bacteria/phage cocktails can affect the larval gut microbiota, thereby affecting their metabolites. The upregulation of beneficial metabolites, such as lactate, taurine, and D-glutamine, in the BB group might promote larval development. Conversely, some beneficial metabolites, such as linoleic acid, taurine, D- and L-glutamine, were downregulated in the other groups. The results revealed that alterations in the metabolites significantly influenced larval development. The development of *Drosophila* larvae requires a rapid conversion of nutrients into biomass, achieved by increasing carbohydrate metabolism and lactate dehydrogenase (LDH) activity [34]. In one study, the survival of the nymphal and premating adult and egg viability were significantly increased in both taurine-containing treatments [50]. Taurine also increases the fly’s tolerance to noxious environmental stimuli [51]. Blocking the activity of glutamine synthetase in the oriental fruit fly (*Bactrocera dorsalis*) decreased glutamine synthesis, which inhibited larval development [52]. Taken together, changes in certain metabolites affect the growth and reproduction of insects, which further supports our conclusion.

Chemical insecticides are the most widely used strategy for controlling disease-carrying pests. However, an excessive use of some chemical insecticides may create environmental concerns and resistance to insecticide, and these concerns are driving research on safer alternatives for pest and vector insect control. Most focus has been on entomopathogenic fungi for use in microbial control due to their potential as biological control agents for pests [53–55]. The potential of bacterial pathogens has received limited attention. The impact of an entomopathogenic fungi treatment may be more persistent and thereby result in longer-term control; however, bacteria are faster acting than fungi once they gain entry to the host. *S. marcescens* as a common natural bioinsecticide is pathogenic to insects [29, 56]. *Bacillus thuringiensis* has different proteins that are toxic to a variety of insects [57], which will provide various alternatives for insect control and for overcoming the pesticides resistance [58]. The entomopathogenic effect of the combined action of different microorganisms, such as *Beauveria bassiana* and *Bacillus thuringiensis var. israelensis*, against the housefly will be greater than that of a single microorganism [26]. The various pathogen combinations as potential alternative methods for the biological control of pests need to be studied further. Thus, the purpose of this study was to test the efficacy of bacteria and bacteriophage cocktails for biological control of houseflies. Bacteria and bacteriophage cocktails are more disruptive on larval gut microbiota and thus inhibit larval growth. The use of pathogens for the control of insects has several advantages that include no resistance, no pollution of the environment and safety for humans. Therefore, microbial insecticides are becoming increasingly important tools in pest management.

## Conclusions

The larval gut contains complex symbiotic microbiota that affect the host’s physiological functions. Our findings showed that the commensal bacteria in the larval gut influenced larval growth. Harmful bacteria/bacteriophage cocktails can be used to develop novel biological control strategies for pests. The growth of the housefly population can be controlled by regulating intestinal microecology. Notably, our previous results revealed that oral administration of a single bacteriophage shows ideal effects on gut microbial regulation. However, oral administration of beneficial/harmful bacteria phage cocktails inhibited larval growth, possibly because the effects of bacteriophage cocktails are more disruptive on larval gut microbiota, increasing harmful bacteria, and thus inhibiting larval growth. Therefore, both beneficial and harmful bacteriophage cocktails will help to develop a new environment-friendly strategy to control harmful insects. This study provides novel insights for the biological control of pests.

### Supplementary Information

**Additional file 1: Fig. S1.** Annotated genome maps of seven kinds of phages. In the circular genome map, the outermost black circle represents the full length of the genome, the innermost multicolored circle represents annotated functional proteins, the second outermost purple circle represents GC content, the third outermost green circle represents GC skew, and the fourth outermost grey circle represents hypothetical proteins.

**Additional file 2: Fig. S2.** Antagonism experiment comparing Ef and cultivable bacteria in the housefly larval intestine. Antagonism experiment comparing Ef and cultivable bacteria, including EhX, Ec in an aerobic environment in the first two pictures. Antagonism experiment comparing Ef and cultivable bacteria, including EhX, Ec in an anaerobic environment in the last two pictures. EhX: *E. hormaechei* EhX; Ec: *E. cloacae* Ec. Data are shown as the means ± SEMs. The t-test was used for the statistical analysis.

**Additional file 3: Fig. S3.** Microbiota-metabolites correlation network based on Spearman’s correlation coefficients.

## Data Availability

The phage sequences were deposited in NCBI (Pkc: OQ884026, Pec: OQ884028, Ppc: OQ884027, Pbc: OQ884030, Phc: MZ669808 [35], Pfc: OQ884029, EfP: OP889240). The 16 s sequence data of the microbiome were stored in the Sequence Read Archive database (BioProject accession number: PRJNA971253).

## Abbreviations

16S rRNA: 16S ribosomal RNA
OTU: Operational taxonomic unit
