# Supplementary material for: Application of bacteria and bacteriophage cocktails for biological control of houseflies
Source: Parasit Vectors. 2024 Jan 17;17:22. doi: 10.1186/s13071-023-06082-8 (PMC10795258; doi:10.1186/s13071-023-06082-8)
Supplement: Supplementary file 1 — Additional file 1: Fig. S1. Annotated genome maps of seven kinds of phages. In the circular genome map, the outermost black circle represents the full length of the genome, the innermost multicolored circle represents annotated functional proteins, the second outermost purple circle represents GC content, the third outermost green circle represents GC skew, and the fourth outermost grey circle represents hypothetical proteins. [file 13071_2023_6082_MOESM1_ESM.docx]

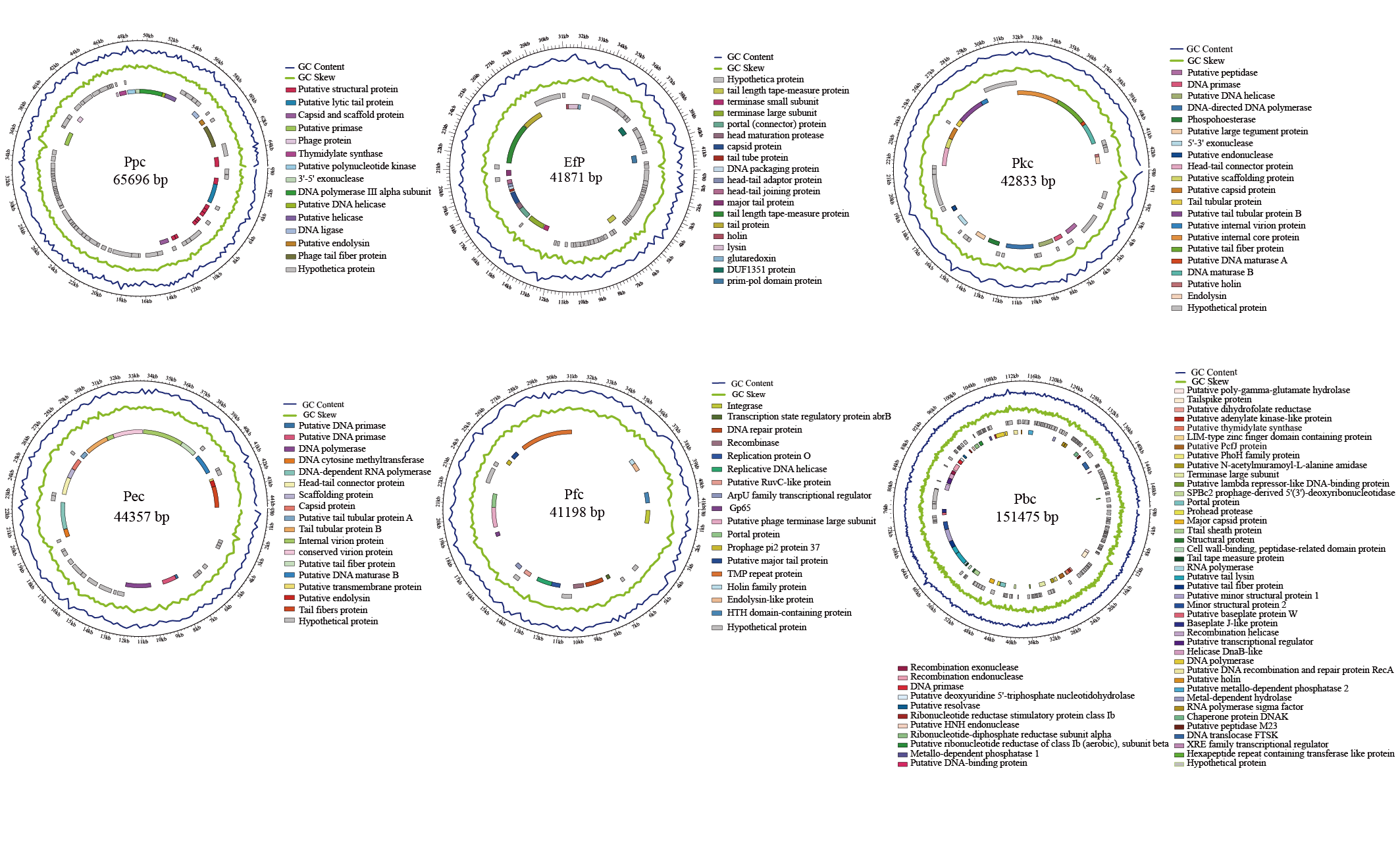


**Fig. S1. Annotated genome maps of seven kinds of phages.** In the circular genome map, the outermost black circle represents the full length of the genome, the innermost multicoloured circle represents annotated functional proteins, the second outermost purple circle represents GC content, the third outermost green circle represents GC skew, and the fourth outermost grey circle represents hypothetical proteins.
