# Supplementary material for: Application of bacteria and bacteriophage cocktails for biological control of houseflies
Source: Parasit Vectors. 2024 Jan 17;17:22. doi: 10.1186/s13071-023-06082-8 (PMC10795258; doi:10.1186/s13071-023-06082-8)
Supplement: Supplementary file 2 — Additional file 2: Fig. S2. Antagonism experiment comparing Ef and cultivable bacteria in the housefly larval intestine. Antagonism experiment comparing Ef and cultivable bacteria, including EhX, Ec in an aerobic environment in the first two pictures. Antagonism experiment comparing Ef and cultivable bacteria, including EhX, Ec in an anaerobic environment in the last two pictures. EhX: E. hormaechei EhX; Ec: E. cloacae Ec. Data are shown as the means ± SEMs. The t-test was used for the statistical analysis. [file 13071_2023_6082_MOESM2_ESM.docx]

**
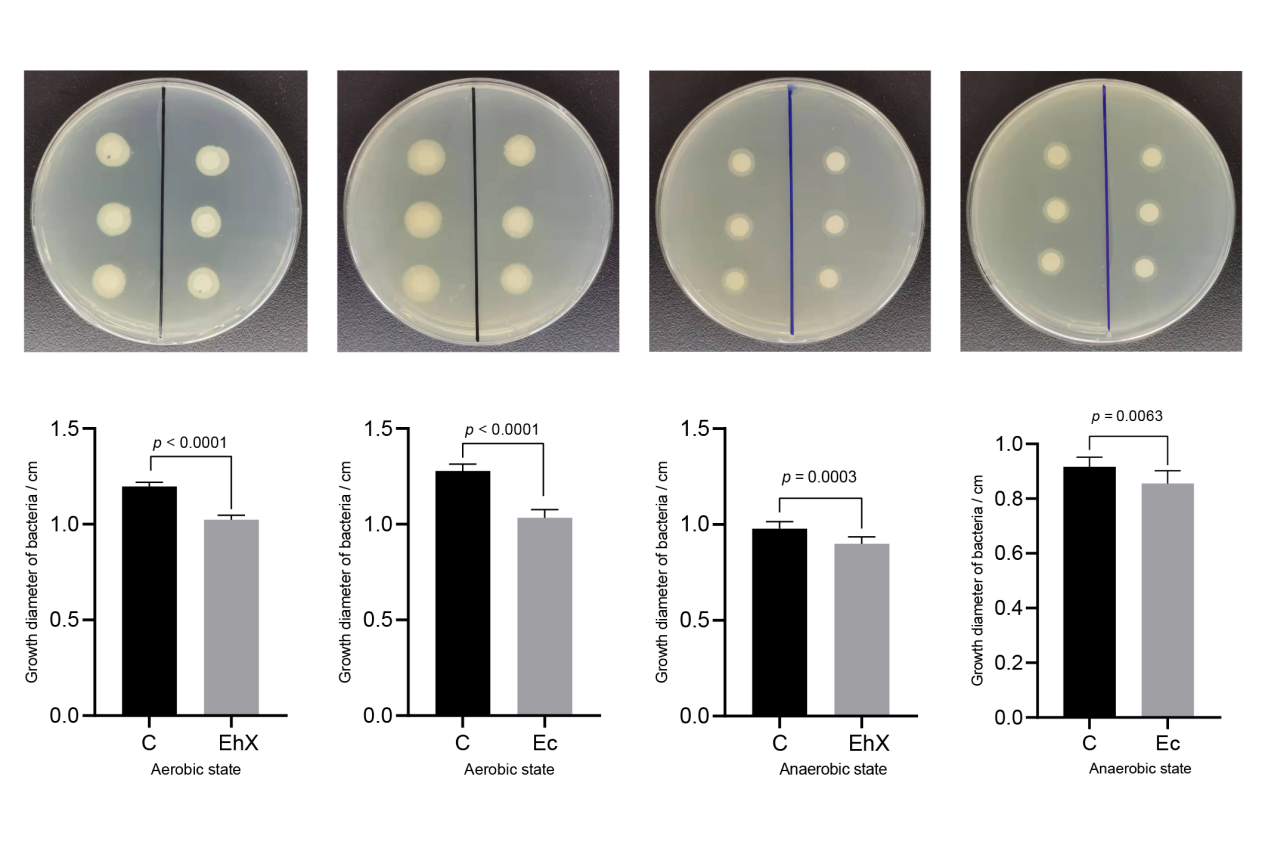
**

**Fig. S2.** Antagonism experiment comparing *E. faecalis* Ef and cultivable bacteria in the housefly larval intestine. Antagonism experiment comparing *E. faecalis* Ef and cultivable bacteria, including *E. hormaechei* EhX, *E. cloacae* Ec in an aerobic environment in the first two pictures. Antagonism experiment comparing *E. faecalis* Ef and cultivable bacteria, including *E. hormaechei* EhX, *E. cloacae* Ec in an anaerobic environment in the last two pictures. EhX: *E. hormaechei* EhX；Ec: *E. cloacae* Ec. Data are shown as the means ± SEMs. The t test was used for the statistical analysis.
