# Supplementary material for: Application of bacteria and bacteriophage cocktails for biological control of houseflies
Source: Parasit Vectors. 2024 Jan 17;17:22. doi: 10.1186/s13071-023-06082-8 (PMC10795258; doi:10.1186/s13071-023-06082-8)
Supplement: Supplementary file 3 — Additional file 3: Fig. S3. Microbiota-metabolites correlation network based on Spearman’s correlation coefficients. [file 13071_2023_6082_MOESM3_ESM.docx]

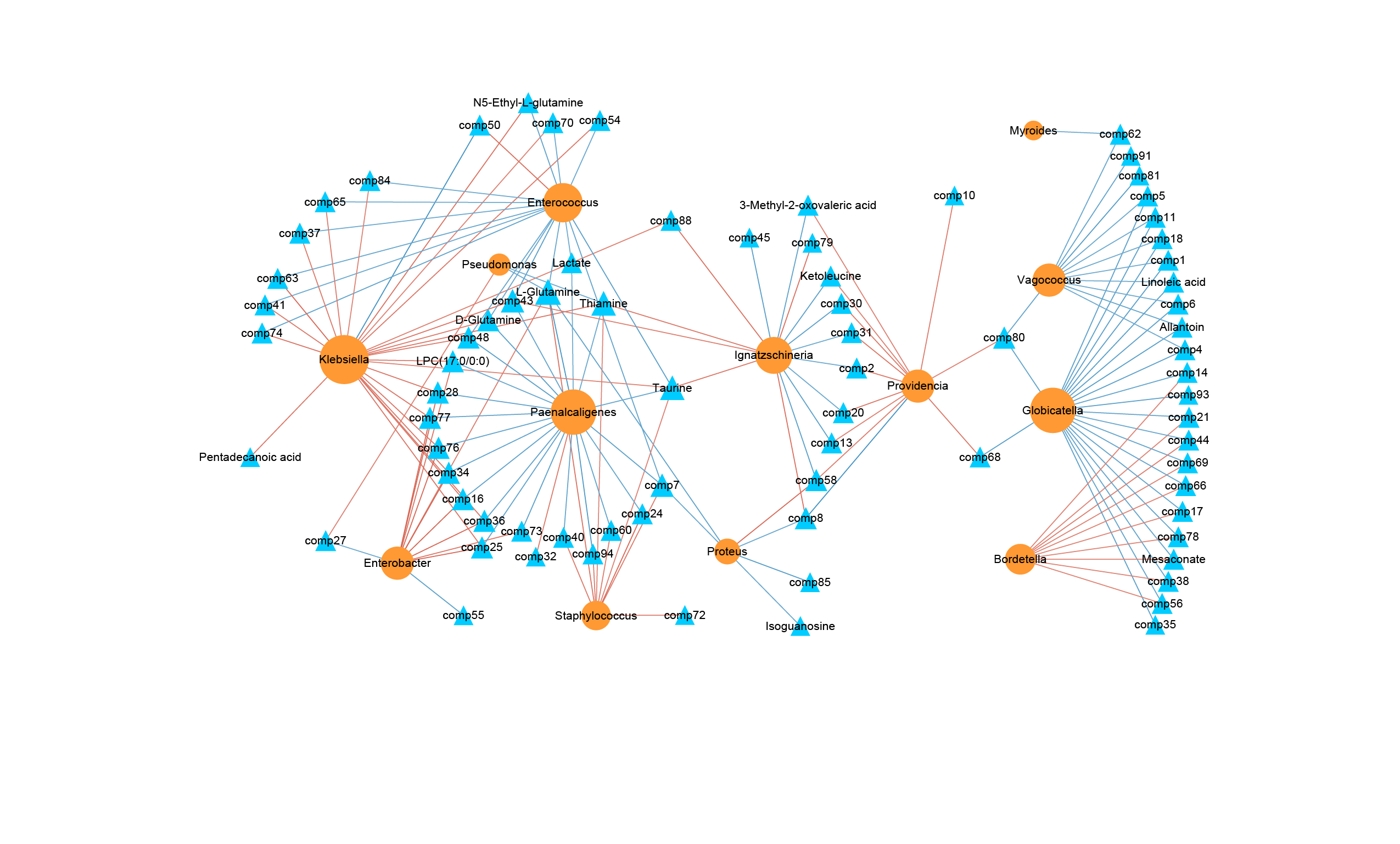


**Fig. S3.** Microbiota-metabolites correlation network based on Spearman’s correlation coefficients.
